# ATM inhibition enhances Auranofin-induced oxidative stress and cell death in lung cell lines

**DOI:** 10.1371/journal.pone.0244060

**Published:** 2020-12-18

**Authors:** Vanessa Ehrenfeld, Jan R. Heusel, Simone Fulda, Sjoerd J. L. van Wijk

**Affiliations:** 1 Institute for Experimental Cancer Research in Pediatrics, Goethe-University Frankfurt, Frankfurt, Germany; 2 German Cancer Consortium (DKTK), Partner Site Frankfurt, Frankfurt, Germany; 3 German Cancer Research Centre (DKFZ), Heidelberg, Germany; Universidade de Sao Paulo Instituto de Biociencias, BRAZIL

## Abstract

Ataxia-Telangiectasia (A-T), a pleiotropic chromosomal breakage syndrome, is caused by the loss of the kinase Ataxia-telangiectasia mutated (ATM). ATM is not only involved in the response to DNA damage, but also in sensing and counteracting oxidative stress. Since a disturbed redox balance has been implicated in the pathophysiology of A-T lung disease, we aimed to further explore the interplay between ATM and oxidative stress in lung cells. Using a kinetic trapping approach, we could demonstrate an interaction between the trapping mutant TRX1-CS and ATM upon oxidative stress. We could further show that combined inhibition of thioredoxin reductase (TrxR) and ATM kinase activity, using Auranofin and KU55933 respectively, induced an increase in cellular reactive oxygen species (ROS) levels and protein oxidation in lung cells. Furthermore, ATM inhibition sensitized lung cells to Auranofin-induced cell death that could be rescued by ROS scavengers. As a consequence, targeted reduction of ATM by TRX1 could serve as a regulator of oxidative ATM activation and contribute to the maintenance of the cellular redox homeostasis. These results highlight the importance of the redox-active function of ATM in preventing ROS accumulation and cell death in lung cells.

## Introduction

The serine/threonine kinase ATM is involved in sensing DNA damage upon DNA double-strand breaks (DSBs) and subsequent activation of cell cycle checkpoints [1–3]. ATM belongs to the phosphoinositide 3-kinase-related kinase (PIKK) family [1, 3]. Other members of this family include ATM and Rad3-related-protein kinase (ATR), DNA-dependent protein kinase catalytic subunit (DNA-PKcs) and mammalian target of rapamycin (mTOR) [1, 2].

Biallelic loss of the *ATM* gene causes A-T, a pleiotropic chromosomal breakage syndrome [4–6]. A-T patients suffer from progressive cerebellar degeneration, ocular telangiectasia, immunodeficiency, genomic instability and increased susceptibility for lymphoid cancer [6, 7]. Respiratory failure upon severe recurrent pulmonary infections and interstitial pulmonary fibrosis is one of the major causes for mortality in A-T [7–9].

ATM is recruited to DNA DSBs through the MRE11-RAD50-NBS1 (MRN) complex [10]. Activation of ATM involves autophosphorylation at S1981 and dissociation of the inactive dimer to the active monomers [11]. Upon DNA damage, ATM phosphorylates a plethora of substrates including histone H2AX, transcription factor p53 and checkpoint kinase 2 (CHK2), thereby activating various signaling networks [12–16].

ATM also plays a role in sensing and counteracting oxidative stress; loss of this action is considered to contribute to A-T pathophysiology [4, 17, 18]. Indeed, a role for ATM as a sensor for oxidative stress has been proposed, since elevated ROS levels and vulnerability to oxidizing agents are characteristics of ATM-deficient cells [19–21]. Oxidative stress directly activates ATM and, upon oxidation, ATM undergoes disulfide-linked dimerization, leading to autophosphorylation at S1981 [17]. ROS-mediated ATM activation is independent of DNA DSBs and the MRN complex, but relies on the oxidation of ATM C2991 [17, 22]. In addition, ATM induces the pentose phosphate pathway (PPP) through phosphorylation of heat shock protein 27 (HSP27), thereby enhancing glucose-6-phosphate dehydrogenase (G6PD) activity [23]. The PPP is the major cellular source for nicotinamide adenine dinucleotide phosphate (NADPH) and the nucleotide precursor ribose-5-phosphate required for DNA repair [23, 24]. Furthermore, NADPH serves as co-factor for the reduction of the oxidized forms of glutathione (GSH) and thioredoxin (TRX), which are the major cellular thiol-dependent antioxidant systems [25]. Interestingly, a recent study has reported a link between mitochondria-derived ROS, ATM and TRX1, implying that mitochondrial ROS stimulate the formation of disulfide-linked ATM dimers which was further enhanced by loss of TRX1 expression [18]. To understand the role of ROS in the A-T lung pathophysiology we aimed to further explore the role of oxidative stress, ATM and the TRX system on cell viability and cell fate in lung cells.

## Materials and methods

### Cell culture and chemicals

Murine lung fibroblasts (MLFs) were kindly provided by Harald von Melchner (Frankfurt am Main, Germany) and A549 cells by Ralf Schubert (Frankfurt am Main, Germany). HEK 293T cells were obtained from the American Type Culture Collection (ATCC). Human cell lines were authenticated by STR profiling (DSMZ, Braunschweig, Germany). All cell lines were continuously monitored for mycoplasma contamination and cultured in DMEM medium (Life technologies Inc., Eggenstein, Germany) supplemented with 10% fetal calf serum (Biochrom, Berlin, Germany), 1% penicillin/streptomycin (Invitrogen, Karlsruhe, Germany). Culture medium for HEK 293T cells also contained 1 mM sodium pyruvate (Invitrogen, Karlsruhe, Germany). Cells were kept at 37 °C and 5% CO_2_. Chemicals were purchased from Sigma-Aldrich (Merck, Darmstadt, Germany) unless otherwise indicated. Auranofin was obtained from Santa Cruz Biotechnology (Santa Cruz, CA, USA), H_2_O_2_ from Carl Roth (Karlsruhe, Germany) and Bleomycin (BLEOCIN, Calbiochem, Merck).

### Determination of ROS production

To determine ROS production, culture medium was discarded and cells were stained at 37 °C for 30 minutes with 5 μM CM-H_2_DCFDA (H_2_DCF) (Invitrogen) or for 10 minutes with 5 μM MitoSOX (Invitrogen) in white RPMI (Life Technologies Inc.). Cells were detached by trypsination, washed once with PBS, resuspended in white RPMI, put on ice and immediately analyzed by flow cytometry.

### Western blot analysis

Western blot analysis was performed as described previously [26] using the following antibodies: rabbit anti-ATM (Cell Signaling Technology, Beverly, MA, USA), mouse anti-p-ATM (Ser1981) (Cell Signaling Technology), rabbit anti-CHK2 (Cell Signaling Technology), rabbit anti-p-CHK2 (Thr68) (Cell Signaling Technology), rabbit anti-H2AX (Abcam, Cambridge, MA, USA), rabbit anti-γ-H2AX (Novus Biologicals, Centennial, CO, USA), mouse anti-Vinculin (Merck), mouse anti-β-Actin (Santa Cruz Biotechnology). For detection, goat anti-mouse or goat anti-rabbit IgG conjugated to horseradish peroxidase (Santa Cruz Biotechnology) and enhanced chemiluminescence (Amersham Biosciences, Freiburg, Germany) were used. For analysis of PRDX dimer formation, cells were washed twice with PBS (Thermo Fisher Scientific, Waltham, MA, USA) supplemented with 50 mM N-ethylmaleimeide (PBS-NEM) (Sigma-Aldrich) and lysed in CHAPS lysis buffer (10 mM HEPES, pH 7.4; 150 mM NaCl; 1% CHAPS) supplemented with 100 mM NEM and protease inhibitor cocktail (Roche Diagnostics, Mannheim, Germany). For non-reducing conditions, lysates were boiled in non-reducing sample buffer, whereas lysates were boiled in sample buffer containing dithiothreitol (DTT) (Carl Roth) for reducing conditions. Peroxiredoxin (PRDX) dimers were analyzed by SDS-PAGE and Western blotting using the following antibodies: rabbit anti-PRDX1 (Abcam) and rabbit anti-PRDX3 (Abfrontier, Seoul, South Korea).

### Determination of cell viability and cell death

Cell viability was measured using MTT (3-(4,5-dimethylthiazol-2-yl)-2,5-diphenyltetrazolium bromide) assay according to the manufacturer’s instructions (Roche Diagnostics). Cell death was determined by propidium iodide (PI)/Hoechst (Sigma-Aldrich) staining using ImageXpress Micro XLS system and MetaXpress Software (Molecular Devices, Biberach an der Riss, Germany) as described previously [27].

### TRX1 trapping

Trapping constructs pQE60-TRX1-CC, pQE60-TRX1-CS and pQE60-TRX1-SS (each containing a streptavidin-binding peptide and a hexahistidine tag) were a gift from Tobias Dick (Addgene plasmids #98402, #98401 and #98403, respectively). The TRX1 mutants were cloned into a pcDNA3.1^(+)^ vector (Thermo Fisher Scientific) for expression in mammalian cells. For analysis of TRX1-interacting proteins, HEK 293T cells were transiently transfected with either pcDNA3.1^(+)^-TRX1-CC, -CS or -SS or the corresponding empty vector (EV) using Lipofectamine 2000 (Thermo Fisher Scientific). After 24 hours, an oxidative stimulus was applied, after which cells were washed twice with PBS-NEM and lysed in Triton X buffer (30 mM Tris, pH 7.4; 150 mM NaCl; 10% glycerol; 1% Triton X-100, Protease Inhibitor Cocktail) supplemented with 50 mM NEM. TRX1 and interacting proteins were pulled down overnight at 4 °C using Pierce High Capacity Streptavidin Agarose (Thermo Fisher Scientific). Bound protein was eluted by boiling with sample buffer and analyzed using Western blotting and mouse anti-His probe (Sana Cruz Biotechnology).

### Statistical analysis

Results are shown as mean +/- SD. Statistical significance was calculated using one-way ANOVA followed by Tukey’s multiple comparisons test using GraphPad Prism 8.3.1 (GraphPad Software, San Diego, CA, USA). P-values were assigned as follows: * p < 0.5; ** p < 0.01; *** p < 0.001.

## Results

### Kinetic trapping of TRX1 with ATM

**Fig 1:**
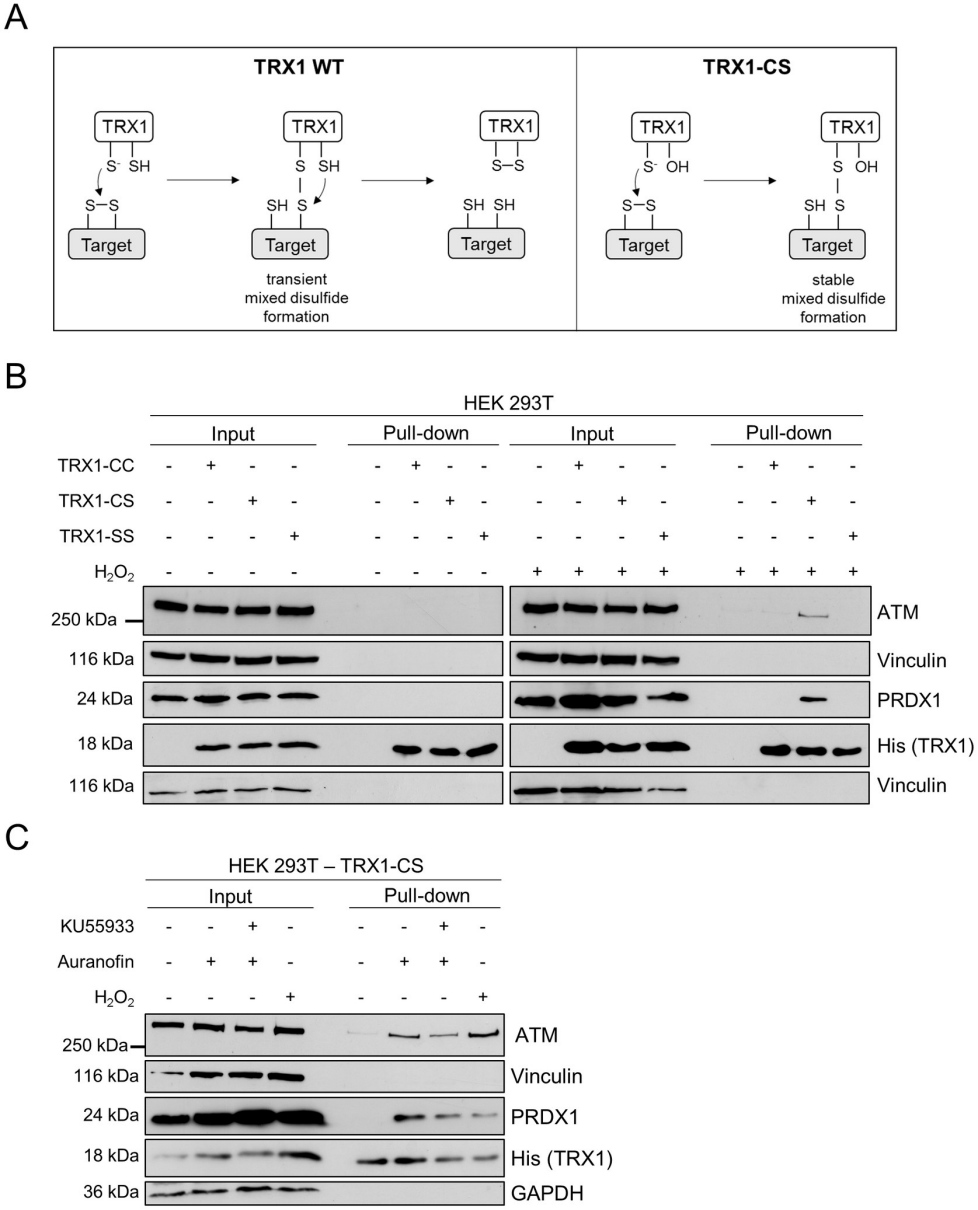
Oxidative stimuli induce an interaction between TRX1-CS and ATM. (A) Schematic representation of the reaction mechanism of the TRX1-mediated reduction of a disulfide, in which WT TRX1 forms a transient mixed disulfide with a target that becomes rapidly resolved, resulting in the reduction of the target protein and TRX1 oxidation. In contrast, the TRX1 trapping mutant (CS), in which the resolving cysteine (C) is mutated to serine (S), cannot dissolve the mixed disulfide resulting in the stabilization of the TRX1-target intermediate. (B) HEK 293T cells expressing TRX1-CC, TRX1-CS or TRX1-SS were subjected to 10 mM H_2_O_2_ for 15 minutes or left untreated. Lysates were prepared in the presence of NEM and TRX1 and proteins were enriched using streptavidin-coated beads. Bound proteins were analyzed by SDS-PAGE and Western blotting. Blots were probed for ATM, PRDX1 and His-tagged TRX1. Vinculin served as loading control. Representative blots of two independent experiments are shown. (C) HEK 293T cells expressing TRX1-CS were subjected to KU55933 (10 μM, 1 hour pre-treatment), Auranofin (4 μM, 4 hours) or H_2_O_2_ (10 mM, 15 minutes) or left untreated. Experimental procedures were similar as described in (B). Vinculin and GAPDH served as loading controls. Representative blots of two independent experiments are shown.

A role for oxidative stress in A-T lung disease has been confirmed in lung cells derived from *Atm*-deficient mice that were highly sensitive to the ROS-inducing DNA-damaging agent Bleomycin [28]. To further understand the functional link between ATM and the TRX system and the relationship between TRX1 levels and ATM oxidation [18], we analyzed a potential direct interaction between TRX1 and ATM. TRX1 reduces oxidized proteins in a two-step mechanism, involving a transient covalent bond between TRX1 and its substrate. Therefore, a previously described kinetic trapping approach was applied, in which TRX1-target disulfide exchange reactions can be monitored [29, 30]. For this, the TRX1 trapping mutant (TRX1-CS), in which the cysteine residue responsible for resolving the covalent bond is mutated to serine (C35S), was used to stabilize the transient TRX1-target interaction (Fig 1A). TRX1-CC served as the corresponding wild-type (WT) control, whereas TRX1-SS (C32S, C35S) acted as a negative control, since this mutant has lost the ability to interact with its target. The remaining three cysteines of TRX1 were mutated to alanines to prevent interference with the trapping reactions [30]. Upon overexpression of the TRX1 mutants in HEK 293T cells, followed by application of an oxidative stimulus, we performed pull-downs of TRX1 as well as associated, trapped TRX-1 targets. By doing so, we detected an interaction between TRX1-CS and the previously described TRX1 target peroxiredoxin 1 (PRDX1) [30] (Fig 1B). Importantly, this interaction was not observed in unstimulated cells or in cells expressing the TRX1-CC or TRX1-SS mutants and confirmed the specificity and functionality of the TRX1 trapping mutants.

Next, we investigated whether we could detect an interaction between TRX1 and ATM under oxidative stress conditions. Indeed, upon stimulation with H_2_O_2_, but not under unstimulated conditions, an interaction between TRX1-CS and ATM could be observed. Similar to the interaction observed for TRX1-CS and PRDX1, ATM only interacted with TRX1-CS, but not with TRX1-CC or TRX1-SS (Fig 1B). The ATM-TRX1-CS interaction could also be detected upon treatment with 1 mM H_2_O_2_ (S1A Fig). Importantly, pull-down of PRDX1 and ATM with TRX1-CS could also be observed upon treatment with Auranofin, an inhibitor of TrxR, but not under unstimulated conditions (Fig 1C). This suggests that Auranofin induces a similar oxidative stress response as H_2_O_2_. Furthermore, inhibition of the ATM kinase activity with KU55933 does not affect the interaction between ATM and TRX1-CS upon stimulation with either Auranofin or H_2_O_2_ (Fig 1C and S1B Fig). Using this kinetic trapping approach, we were able to identify the interaction of ATM with the trapping mutant TRX1-CS upon oxidative stimuli.

### ATM inhibition enhances Auranofin-induced ROS levels in lung cell lines

**Fig 2:**
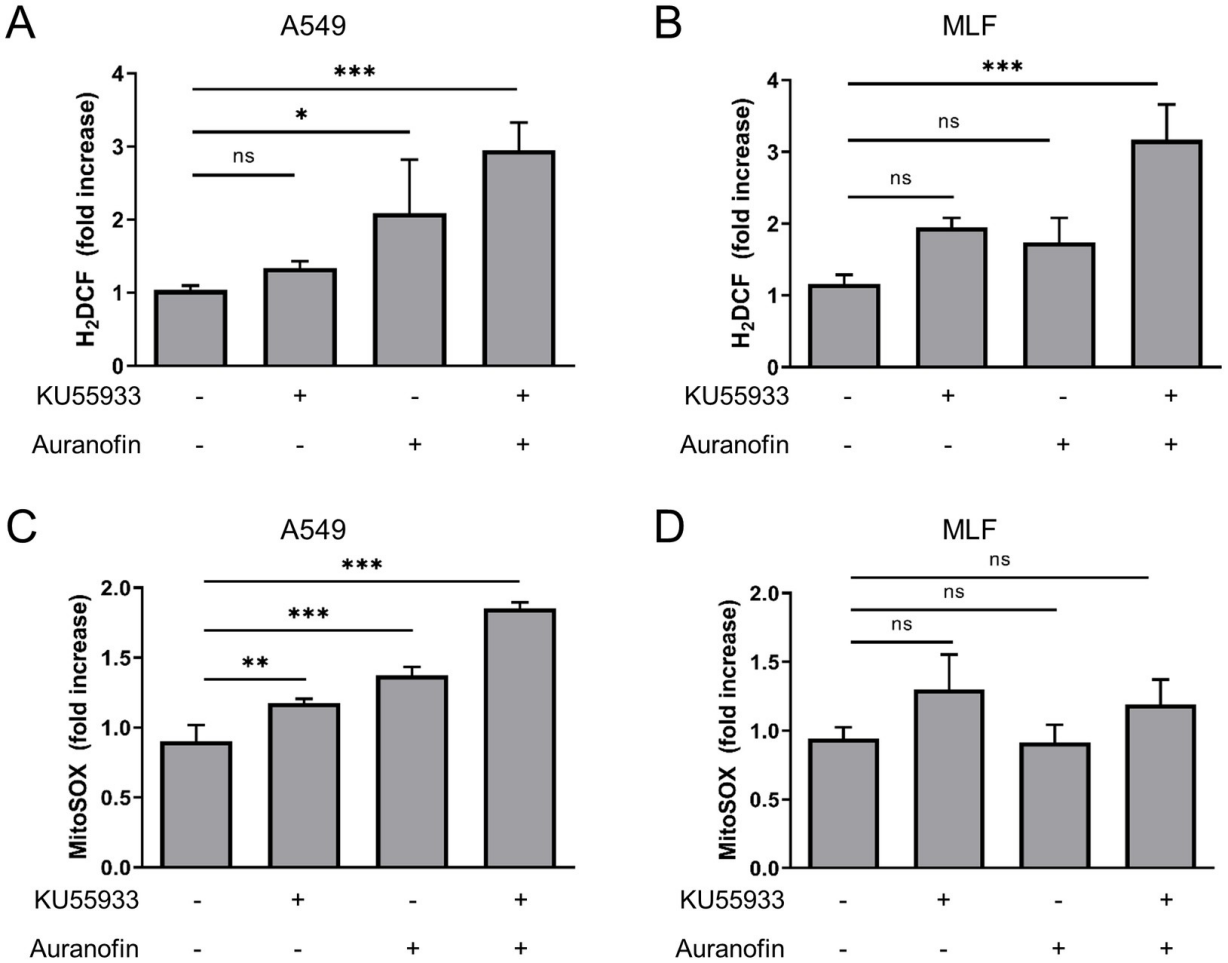
ATM inhibition enhances Auranofin-induced ROS levels in A549 and MLF cells. A549 (A, C) and MLF (B, D) cells were treated as indicated with 10 μM KU55933 for 1 hour prior to treatment with Auranofin (A549: 10 μM, MLF: 2 μM) for 4 hours. Cellular (A, B) or mitochondrial (C, D) ROS levels were measured using the fluorescent dyes H_2_DCF or MitoSOX, respectively, and flow cytometry in A549 (A, C) or MLF cells (B, D). Data were normalized to control. Mean and SD of three independent experiments performed in triplicates are shown. ns: not significant; * p < 0.5; ** p < 0.01; *** p < 0.001.

Oxidative stress has been implicated in the A-T lung pathophysiology [28], but the interplay of ATM inhibition and oxidative stress in lung cells remains unclear. Lung epithelial cells, but not lung fibroblasts, derived from ATM-deficient mice display increased ROS levels [28], therefore, we employed the human lung epithelial cell line A549 and MLFs. Both cell lines were treated with Auranofin followed by quantification of ROS levels (Fig 2A–2D). As MLFs were more sensitive to Auranofin treatment, lower concentrations of Auranofin were used in this cell line. Cellular ROS levels, measured with the fluorescent dye H_2_DCF, as well as mitochondrial ROS levels, quantified with the mitochondria-targeted dye MitoSOX, were significantly increased in A549 cells upon Auranofin treatment (Fig 2A and 2C), but not in MLFs (Fig 2B and 2D). To investigate the role of ATM on Auranofin-induced ROS levels ATM kinase activity was inhibited by KU55933; ATM inhibition alone did already increase ROS levels in A549 cells and MLFs to some extent (Fig 2A–2D). Intriguingly, total cellular ROS levels were increased upon combined ATM kinase and TrxR inhibition in A549 and MLFs (Fig 2A and 2B), whereas mitochondrial ROS increased in A549 cells upon Auranofin/KU55933 co-treatment, compared to untreated cells. As KU55933 treatment alone led to a slight increase in cellular and mitochondrial ROS and significantly elevated ROS levels in combination with Auranofin, these findings suggest that ATM kinase inhibition contributes to the increase in ROS levels upon TrxR inhibition in lung cells.

### Combined TrxR and ATM inhibition induces oxidative dimerization of peroxiredoxins

**Fig 3:**
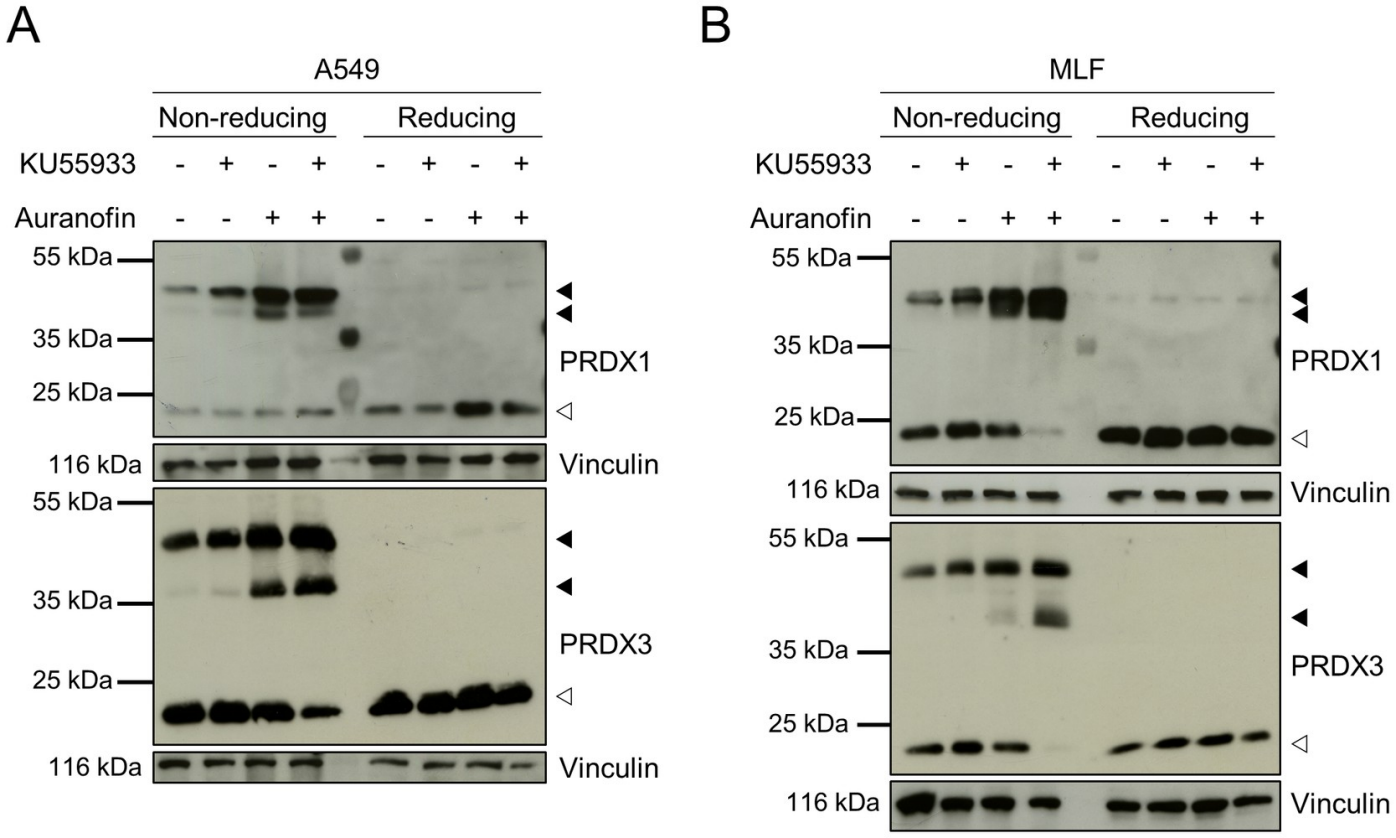
KU55933 and Auranofin induce ROS-dependent dimerization of PRDX1 and PRDX3. A549 (A) and MLF (B) cells were treated as indicated with KU55933 (10 μM, 1 hour pre-treatment) and Auranofin (A549: 10 μM, MLF: 2 μM). Cells were harvested and prepared for SDS-PAGE under non-reducing or reducing conditions. Vinculin was used as loading control. White arrowheads indicate monomeric, filled arrowheads dimeric forms of PRDX1 and PRDX3. Representative blots of two independent experiments are shown.

To further assess the role of ATM kinase and TrxR inhibition in protein oxidation, we analyzed the oxidation status of PRDX1 and -3, two highly oxidation-sensitive proteins, which undergo disulfide-linked dimerization upon oxidation that can be reduced by TRXs [31]. Those disulfide-linked dimers can be visualized on non-reducing SDS-PAGE, in which PRDX dimers appear as multiple bands, since the number of disulfide bonds present in the dimeric species affect gel migration. Dimers with two disulfide bonds are more compact and migrate faster compared to dimers with only one disulfide [32]. Both slow-migrating PRDX1 and PRDX3 dimers could be detected on non-reducing SDS-PAGE upon Auranofin single- and co-treatment with KU55933 in A549 cells and MLFs (Fig 3A and 3B). Importantly, levels of monomeric PRDX1 and -3 decreased upon combined Auranofin and KU55933 treatment, with the exception of PRDX1 in A549 cells. As expected, oxidation-induced dimerization was lost under reducing conditions. Therefore, these results indicate that inhibition of ATM kinase activity promotes PRDX dimerization in Auranofin-treated lung cells.

### Activation of ATM upon Auranofin treatment

**Fig 4:**
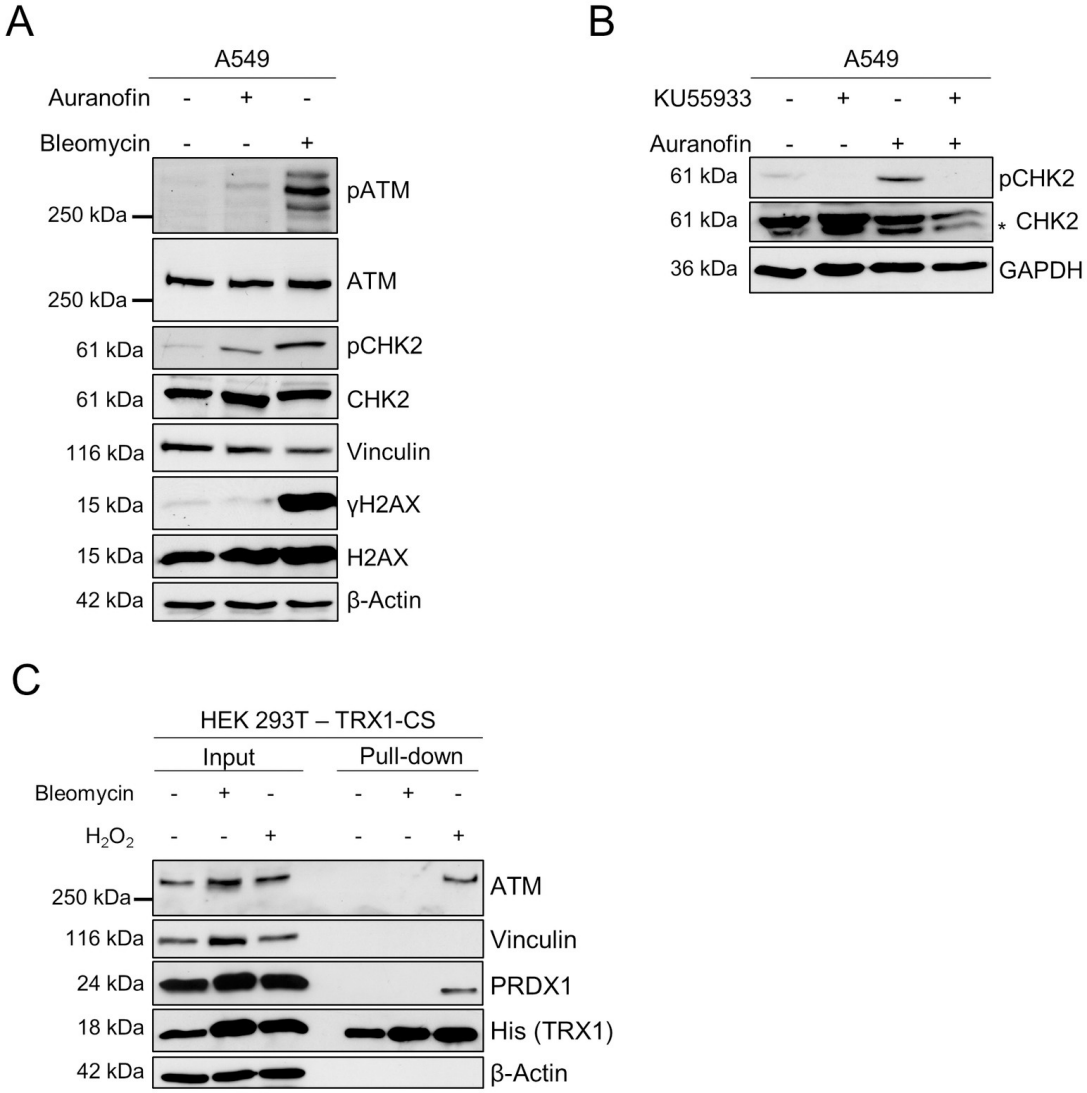
Auranofin induces ATM-dependent phosphorylation of CHK2. (A) A549 cells were treated with Auranofin (10 μM) or Bleomycin (100 μM) for 6 hours or left untreated. Phosphorylation of ATM, CHK2 and H2AX was analyzed by SDS-PAGE and Western blotting. Vinculin and β-Actin served as loading controls. (B) A549 cells were treated as indicated with KU55933 (10 μM, 1 hour pre-treatment) and Auranofin (10 μM, 6 hours). Samples were subjected to SDS-PAGE and Western blotting and CHK2 phosphorylation was assessed with GAPDH as loading control. An unspecific band is marked with an asterisk (*). (C) HEK 293T cells expressing TRX1-CS were subjected to Bleomycin (100 μM, 4 hours) or H_2_O_2_ (10 mM, 15 minutes) or left untreated. Lysates were prepared in the presence of NEM and TRX1 and proteins were enriched using streptavidin-coated beads. Bound proteins were analyzed by SDS-PAGE and Western blotting. Blots were probed for ATM, PRDX1 and His-tagged TRX1. Vinculin and β-Actin served as loading controls. (A, B, C) Representative blots of two independent experiments are shown.

The redox-controlling function of ATM is separated from its role in DNA repair [17, 18, 22]. Since we have detected an ATM-TRX1 interaction upon Auranofin treatment and ATM kinase inhibition increased ROS levels and PRDX dimerization upon Auranofin treatment in lung cells, we analyzed whether ATM becomes activated upon Auranofin treatment. Interestingly, we could detect Auranofin-induced ATM S1991 autophosphorylation, as well as phosphorylation of the ATM target CHK2 on T68, but H2AX phosphorylation (γH2AX), a marker for DNA DSBs, was absent in A549 cells (Fig 4A). In contrast, Bleomycin, an inducer of DNA DSBs, induced phosphorylation of ATM, CHK2 as well as H2AX (Fig 4A). Furthermore, treatment with KU559933 prior to Auranofin completely abrogated CHK2 phosphorylation, confirming that CHK2 phosphorylation is dependent on ATM kinase activity (Fig 4B). Additionally, we performed kinetic trapping experiments upon treatment with Bleomycin to test whether DNA DSBs also induced an ATM-TRX1-CS interaction. Interestingly, pull-down of ATM with TRX1-CS was not observed upon Bleomycin treatment (Fig 4C). These results highlight that Auranofin treatment leads to ATM activation, in the absence of DNA DSBs and possibly through oxidative dimerization, and induces downstream ATM signaling.

### ATM inhibition sensitizes lung cell lines to Auranofin-induced cell death

**Fig 5:**
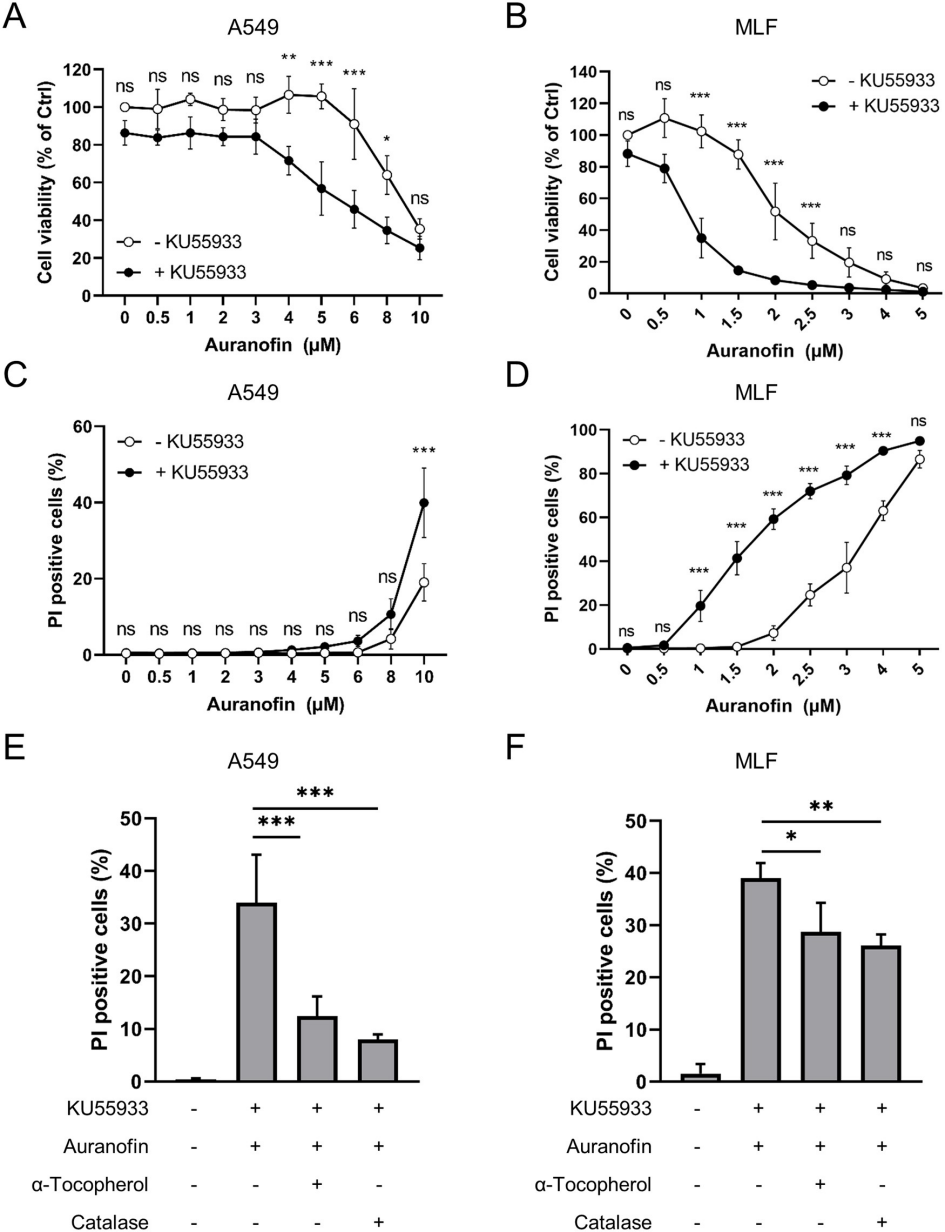
ATM inhibition sensitizes lung cell lines to Auranofin-induced cell death that is ROS-dependent. (A-D) A549 cells (A, C) and MLFs (B, D) were treated with the indicated concentrations of Auranofin in the presence or absence of KU55933 for 48 hours (A549) or 24 hours (MLF). (A, B) Cell viability was analyzed using MTT assay. Data were normalized to control. (C, D) Cell death was assessed by PI/Hoechst staining and fluorescence microscopy. (E, F) A549 cells (E) and MLFs (F) were pre-treated with KU55933 (10 μM) and α-Tocopherol (100 μM) or catalase (10 μg/ml) for 1 hour before addition of Auranofin (A549: 10 μM, MLF: 2 μM). Cell death was assessed after 48 hours (A549) or 24 hours (MLF) using PI/Hoechst staining and fluorescence microscopy. All experiments were performed three times in triplicates. Mean and SD are shown. ns: not significant; * p < 0.5; ** p < 0.01; *** p < 0.001.

To understand how TrxR and ATM inhibition affects cell fate, A549 cells and MLFs were treated with increasing concentrations of Auranofin in the absence and presence of KU55933. Auranofin led to a dose-dependent loss of cell viability in MLFs after 24 hours and in A549 cells after 48 hours (Fig 5A and 5B). Combined ATM kinase inhibition using KU55933 and Auranofin treatment significantly reduced cell viability compared to Auranofin single treatment (Fig 5A and 5B), whereas KU55933 treatment alone had no effect on cell viability. As the quantification of cell viability using the MTT assay does not discriminate between alterations in cell death or cell proliferation, PI stainings were performed (S2 Fig). Auranofin induced dose-dependent cell death in A549 cells and MLFs that was increased when ATM kinase activity was simultaneously inhibited by KU55933 (Fig 5C and 5D). The amount of cell death was inversely correlated with the decrease in cell viability but, interestingly, MLFs were more sensitive to Auranofin treatment compared to A549 cells. To evaluate whether loss of cell viability and induction of cell death were mediated by oxidative stress, cells were treated with the H_2_O_2_ scavenger catalase or the ROS scavenger α-Tocopherol prior to Auranofin and KU55933 treatment. Importantly, both scavengers significantly attenuated Auranofin/KU55933-induced cell death in A549 cells and MLFs (Fig 5E and 5F). With these experiments, we could further emphasize the role of ATM kinase activity in preventing Auranofin-induced cell death.

## Discussion

Here, we demonstrate that lung cells in which the ATM kinase activity was inhibited by KU55933 displayed increased cytosolic and mitochondrial ROS levels upon Auranofin treatment. As a consequence, protein oxidation was increased as demonstrated by the enhanced formation of PRDX1 and PRDX3 dimers. These findings are in line with previous reports of increased levels of ROS and markers of oxidative damage in cells and tissues of ATM-deficient mice and A-T patients [19, 28, 33–35]. Furthermore, we demonstrated that ATM redox signaling is important for cellular survival, since ATM kinase inhibition sensitizes cells for Auranofin-induced cell death. Interestingly, MLFs were more sensitive to Auranofin treatment than A549 cells even though the increase in ROS upon the lower Auranofin concentration was not as pronounced as in A549 cells. As A549 cells are derived from a lung adenocarcinoma [36], it is likely that these cells are less susceptible to stress-inducing treatments than MLFs. Moreover, cell death induced by Auranofin/KU55933 co-treatment could be rescued by catalase or α-Tocopherol, showing the detrimental effects of unbalanced ROS production on cellular survival.

The use of kinetic trapping allowed the detection of an ATM-TRX1-CS interaction upon oxidative stress in HEK 293T cells. TRX1 trapping approaches have been used to identify the interaction between TRX1 and CD30 in mammalian cells [30], as well as the involvement of TRX1 in various cellular pathways in E.coli [37]. Interestingly, TRX1 has been implicated as a negative regulator of ATM dimer formation [18], however, a direct interaction has not yet been reported, as TRX1-target interactions are usually too transient to be detected using regular interaction assays. Our findings underscore the role of ATM as an oxidative stress sensor and support the notion that TRX1 might be involved in a negative feedback loop that controls the redox-active functions of ATM [18]. Further experiments are however required to identify the interaction of ATM with endogenous WT TRX1, to exclude that the interaction is caused by overexpression of the trapping mutant. Furthermore, functional studies are also required to confirm the biological relevance of this interaction. We speculate that TRX1 keeps ATM in the reduced or inactive state when the formation and detoxification of ROS are balanced. However, in situations of oxidative stress, ATM is oxidized and induces antioxidant pathways. Upon restoration of the redox balance, TRX1 can be regenerated, thereby reducing ATM dimers and diminishing its antioxidant signaling. However, the cysteine residues in ATM that are specifically reduced by TRX1 remain to be identified. Importantly, inhibition of ATM kinase activity using KU55933 did not interfere with binding of TRX1-CS to ATM.

Interestingly, an interaction of the trapping mutant TRX1-CS with PRDX1 and ATM could also be observed upon stimulation with TrxR inhibitor Auranofin. Since TRX can only interact with its targets in its reduced state, treatment with Auranofin did not lead to a complete exhaustion of the cellular thioredoxin pool in our system. Previous reports stated an increase in mitochondria-derived H_2_O_2_ after Auranofin treatment [38]. It is possible that this H_2_O_2_ production precedes the oxidation of cytosolic TRX1.

Oxidative stress has long been known to play a key role in the pathogenesis of A-T [20]. Here, we provide further evidence showing that ATM is involved in the antioxidant response to oxidative stress. Our findings recapitulate the redox-active functions of ATM and implications for lung cell fate upon induction of oxidative stress. Our observations further underscore previous findings that link increased ROS levels to lung disease in A-T [28]. Indeed, administration of antioxidants decreases cell death observed upon Auranofin and ATM inhibition. Further experiments are required to evaluate whether systemic or local antioxidant administration could have beneficial effects on the progression of lung disease in A-T models and patients.

Increased ROS levels and oxidative stress are not only involved in damaging lung tissues of A-T patients, but are also linked to cerebellar degeneration and death of Purkinje cells [20, 34]. Additional experiments to understand and elucidate the role of TRX1 in these tissues would contribute to a better understanding of A-T pathology. In addition, increased ROS levels have also been implicated in other neurological diseases, like Parkinson’s disease and Alzheimer’s disease [20, 39, 40] and the TRX system plays important roles in both pathologies [40–42]. It seems likely that the here described TRX1-ATM interaction might also play a role in these pathologies. Future research is required to test this hypothesis and to elucidate whether increasing TRX1 abundancy or regeneration could have beneficial effects on these diseases.

In contrast, increases in ATM signaling have been linked to radiation resistance of breast cancer cells [43], and inhibition of ATM kinase activity using KU55933 sensitized tumor cells to radiation and chemotherapy [44]. Therefore, failure of ATM to activate specific downstream targets could have beneficial effects in different contexts, for example, combination of ATM inhibitors with inducers of oxidative stress as an anticancer strategy.

In conclusion, our findings indicate an important role for the redox-active functions of ATM in counteracting oxidative stress in lung cells and highlight an interaction between TRX1 and ATM that could contribute to the maintenance of the cellular redox balance.

## Supporting information

S1 Fig The ATM-TRX1-CS interaction is dose-dependent and independent of KU55933 treatment. A) HEK 293T cells expressing TRX1-CS were subjected to H_2_O_2_ (0.1 mM, 1mM, 10 mM for 15 minutes) or left untreated. (B) HEK 293T cells expressing TRX1-CS were subjected to KU55933 (10 μM, 1 hour pre-treatment) or H_2_O_2_ (10 mM, 15 minutes) or left untreated. (A, B) Lysates were prepared in the presence of NEM and TRX1 and proteins were enriched using streptavidin-coated beads. Bound proteins were analyzed by SDS-PAGE and Western blotting. Blots were probed for ATM, PRDX1 and His-tagged TRX1. Vinculin and β-Actin served as loading controls. Representative blots of two independent experiments are shown.
(PDF)

S2 Fig Representative images of PI/Hoechst stained MLFs used for cell death analysis in Fig 5D. MLFs were treated for 24 h with Auranofin (3 μM) or left untreated. Cells were stained with PI and Hoechst and analyzed by fluorescent microscopy. Scale bar = 500 μm.
(PDF)
