# Supplementary figures and images for: ATM inhibition enhances Auranofin-induced oxidative stress and cell death in lung cell lines
Source: PLoS One. 2020 Dec 18;15(12):e0244060. doi: 10.1371/journal.pone.0244060 (PMC7748142; doi:10.1371/journal.pone.0244060)

Figure S1

A

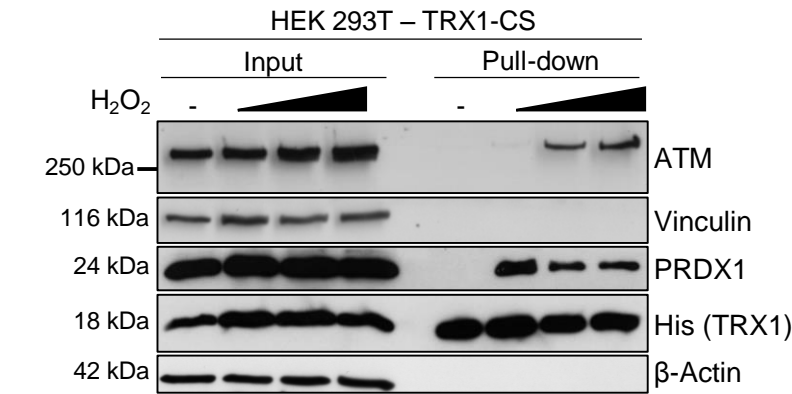

B

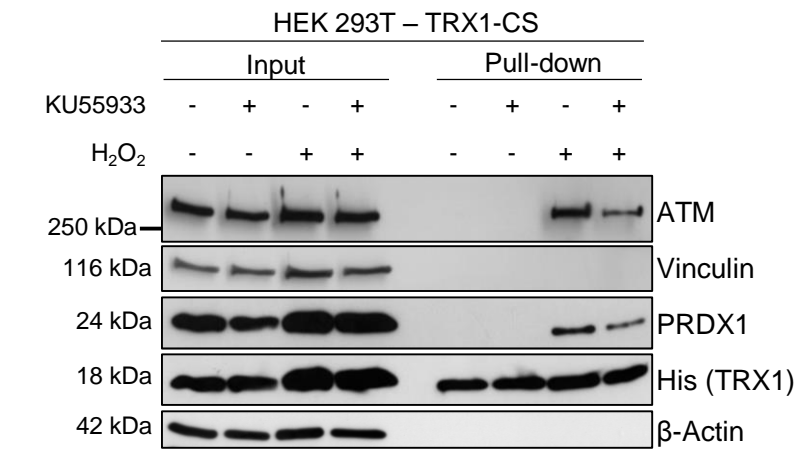

Figure S2

MLF

untreated

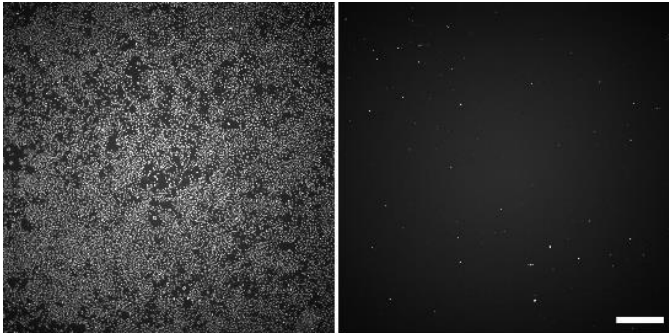

Auranofin (3  $\mu$ M)

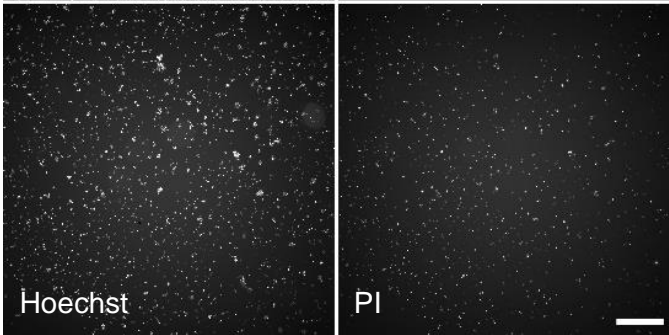

Supplement: S1 Fig — A) HEK 293T cells expressing TRX1-CS were subjected to H2O2 (0.1 mM, 1mM, 10 mM for 15 minutes) or left untreated. (B) HEK 293T cells expressing TRX1-CS were subjected to KU55933 (10 μM, 1 hour pre-treatment) or H2O2 (10 mM, 15 minutes) or left untreated. (A, B) Lysates were prepared in the presence of NEM and TRX1 and proteins were enriched using streptavidin-coated beads. Bound proteins were analyzed by SDS-PAGE and Western blotting. Blots were probed for ATM, PRDX1 and His-tagged TRX1. Vinculin and β-Actin served as loading controls. Representative blots of two independent experiments are shown. (PDF) [file pone.0244060.s001.pdf]
