# Supplementary material for: ATM inhibition enhances Auranofin-induced oxidative stress and cell death in lung cell lines
Source: PLoS One. 2020 Dec 18;15(12):e0244060. doi: 10.1371/journal.pone.0244060 (PMC7748142; doi:10.1371/journal.pone.0244060)

Figure 1 B – scanned original blots

HEK 293T – TRX1-CC/CS/SS -/+ H_2O_2

(1a) ATM

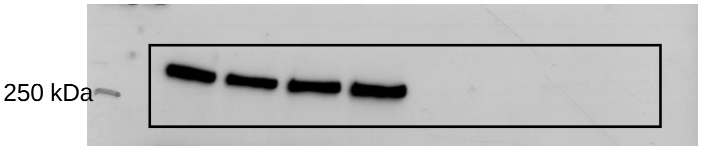

(1b) ATM

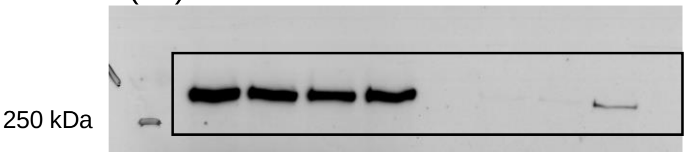

(2a) Vinculin

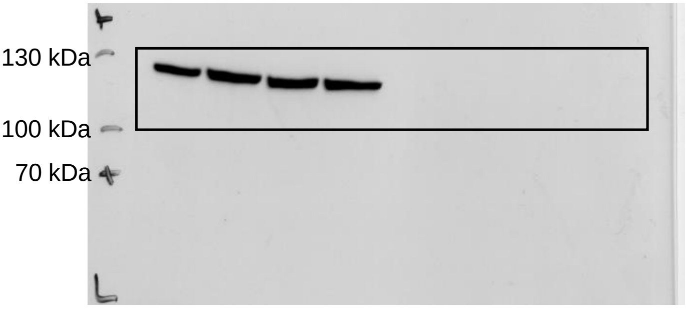

(2b) Vinculin

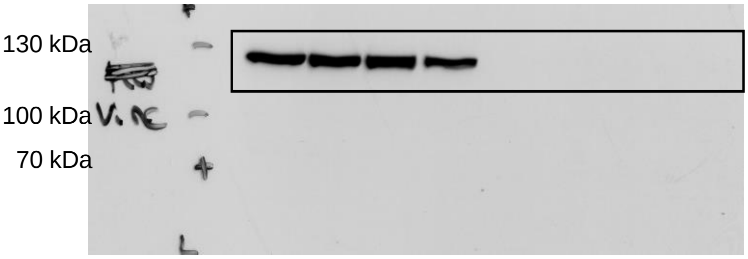

(3a) Vinculin, PRDX1

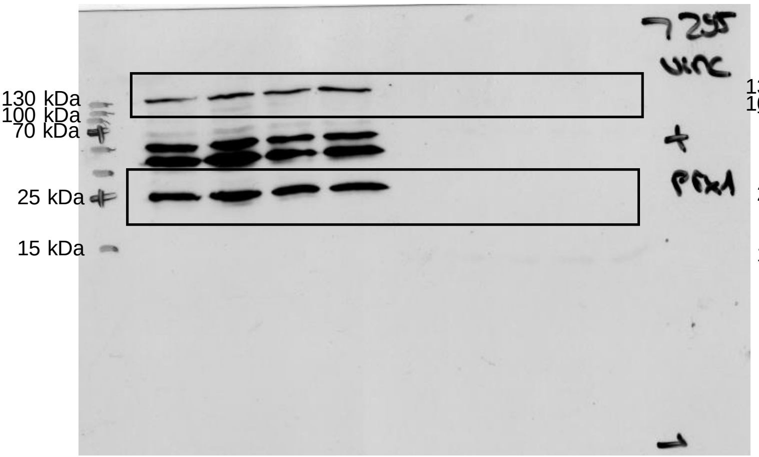

(3b) Vinculin, PRDX1

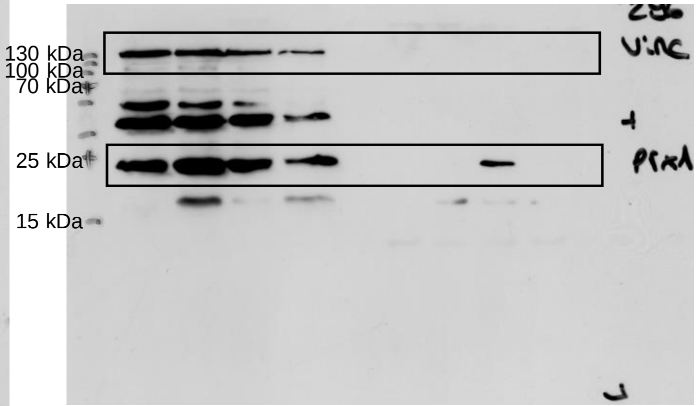

(4a) His

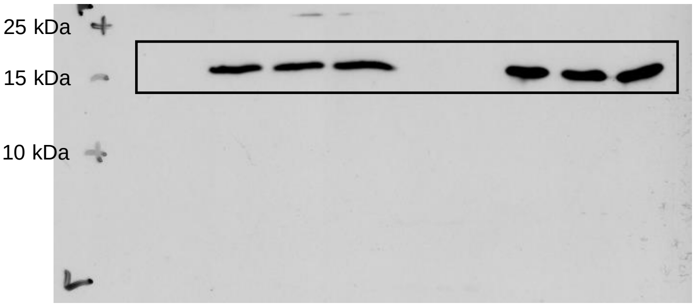

(4b) His

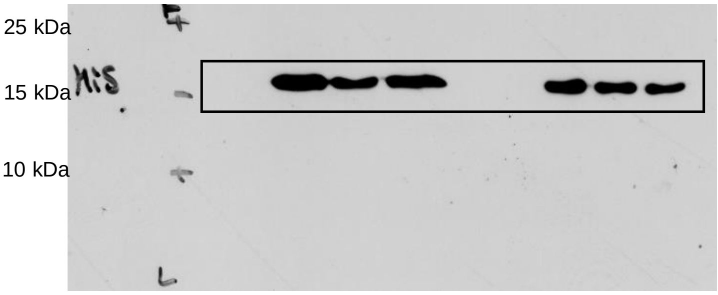

Figure 1 C – scanned original blots

HEK 293T – TRX1-CS -/+ KU55933/Aura/H_2O_2

(1) ATM, Vinculin

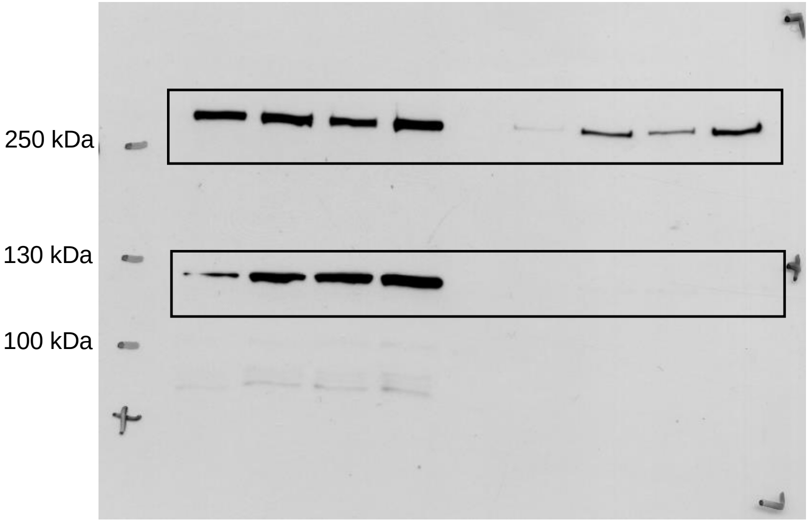

(2) PRDX1

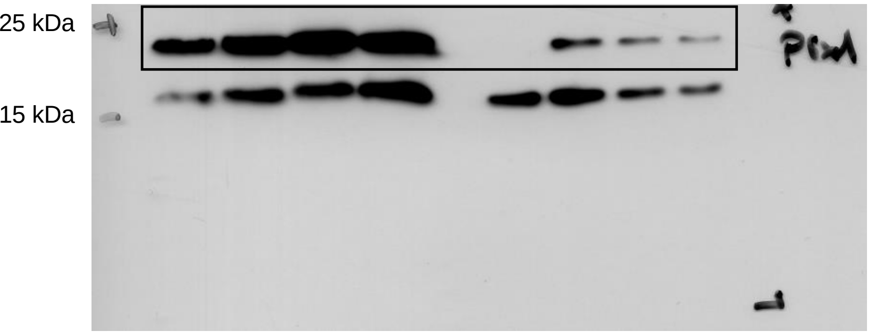

(3) His

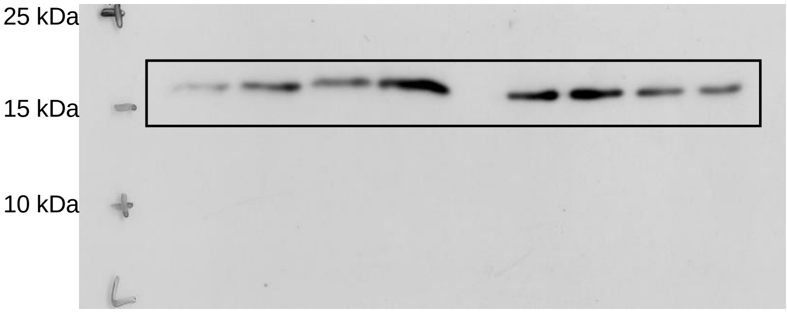

(4) GAPDH

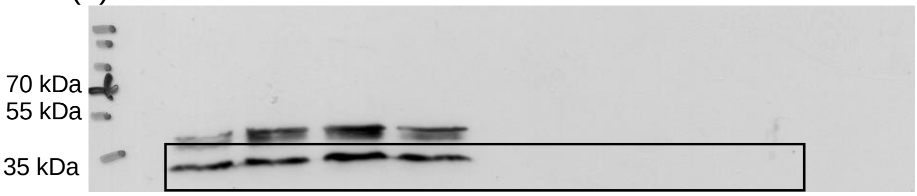

Figure 3 A – scanned original blots

A549 +/- KU55933/Auranofin

(1) PRDX1

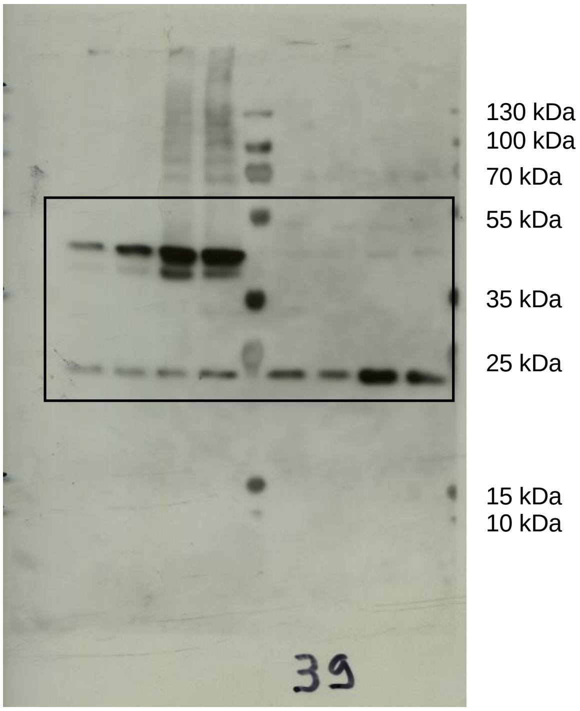

(2) Vinculin

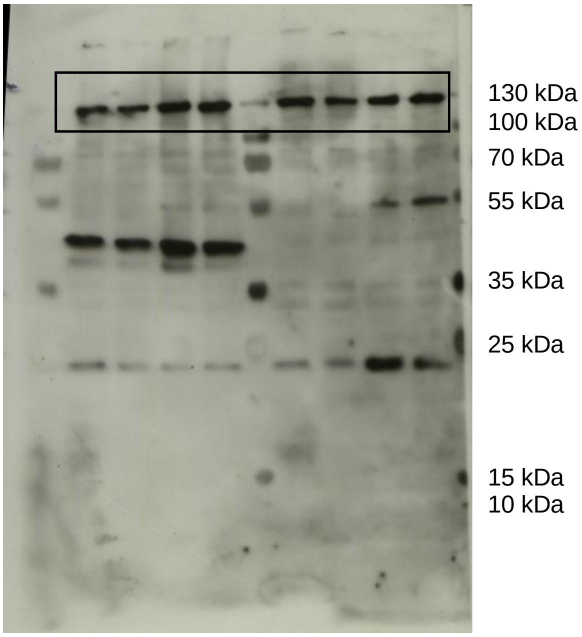

(3) PRDX3

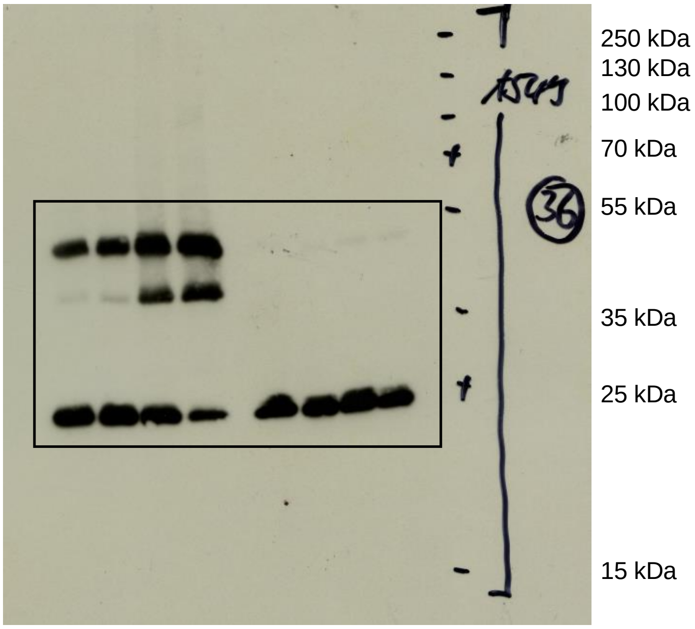

(4) Vinculin

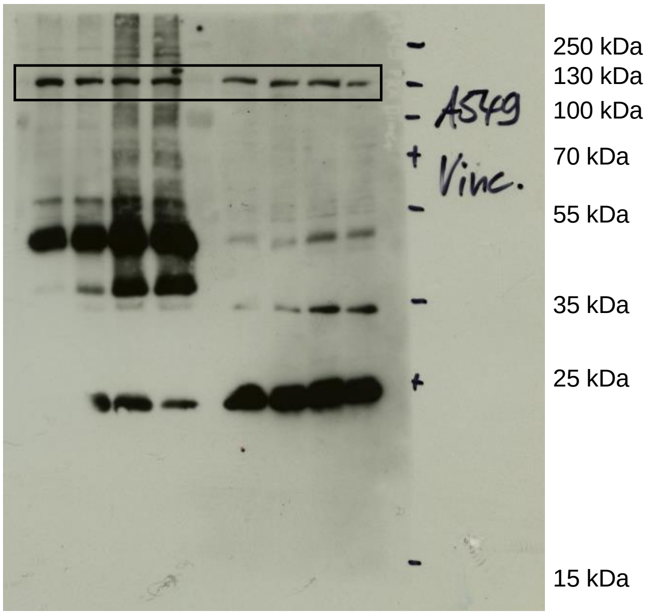

Figure 3 B – scanned original blots

MLF +/- KU55933/Auranofin

(1) PRDX1

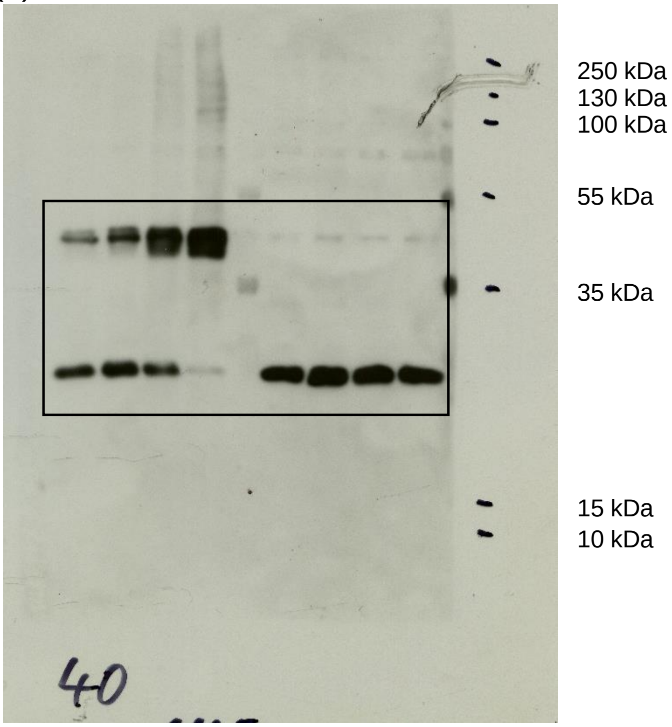

(2) Vinculin

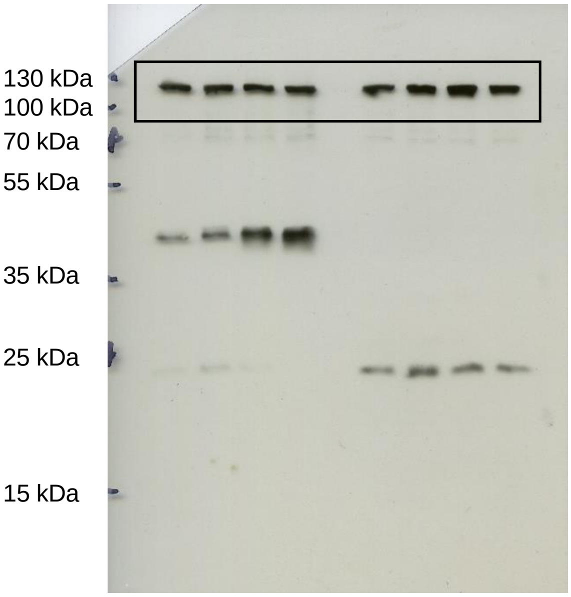

(3) PRDX3

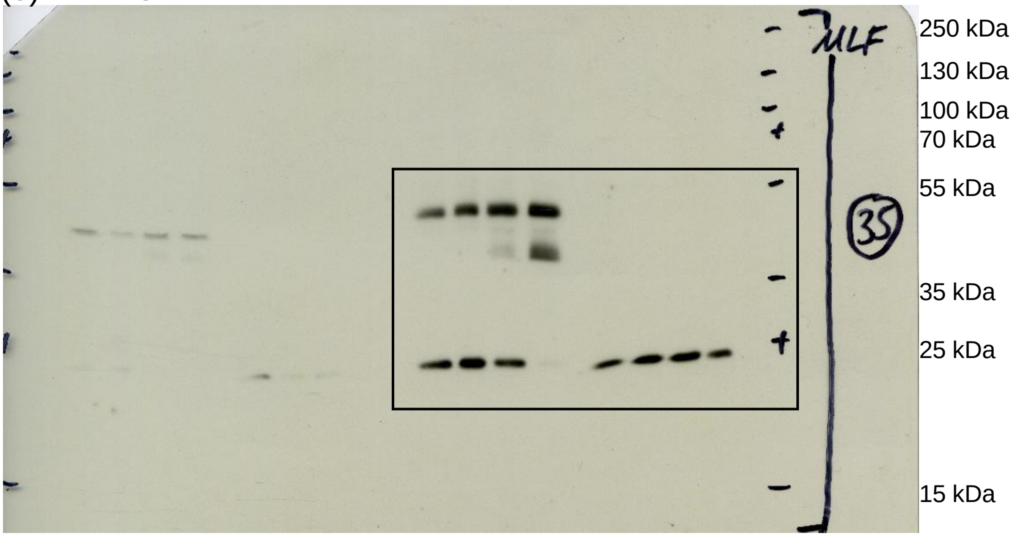

(4) Vinculin

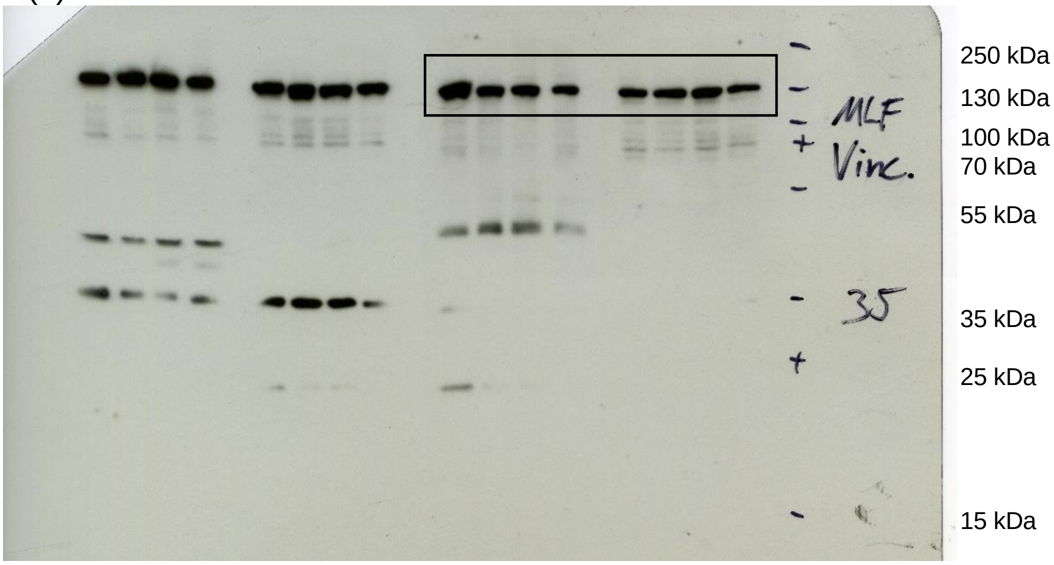

Figure 4 A – scanned original blots

A549 +/- Auranofin/Bleomycin

(1) pATM

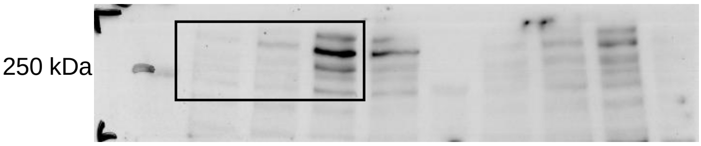

(2) ATM

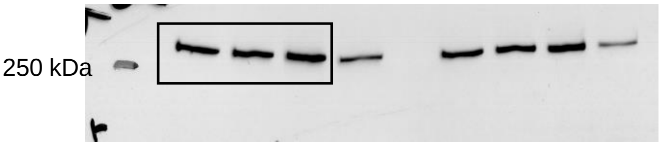

(3) pChk2

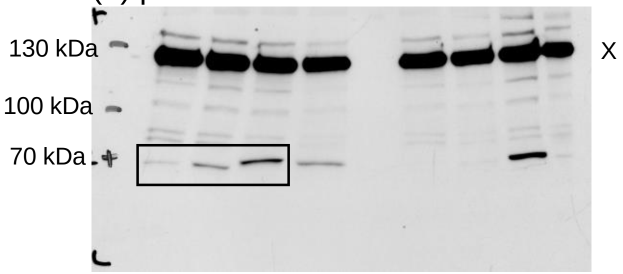

(4) Chk2

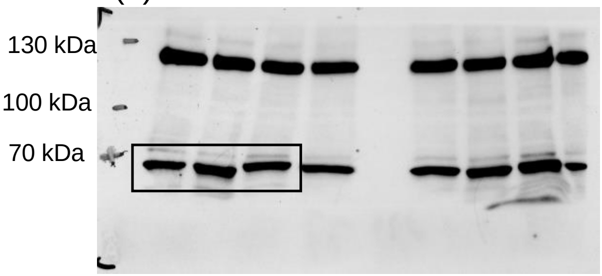

(5) Vinculin

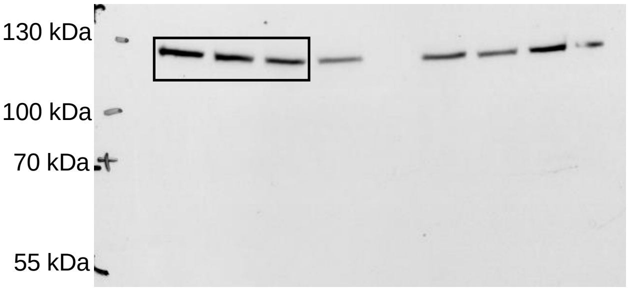

(6) γH2AX

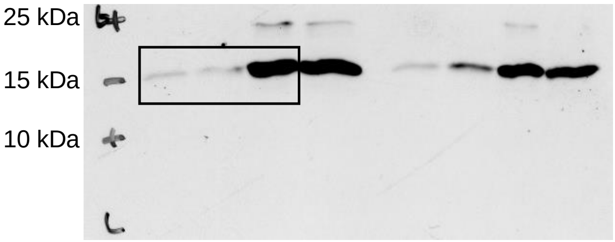

(3) H2AX

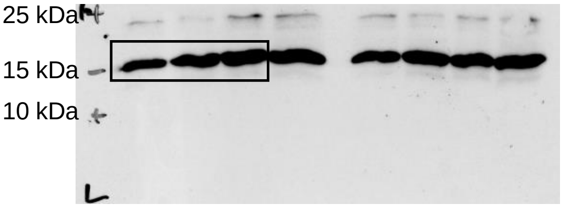

(3) β-Actin

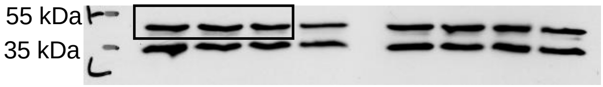

Figure 4 B – scanned original blots

A549 +/- KU55933/Auranofin

(1) pChk2

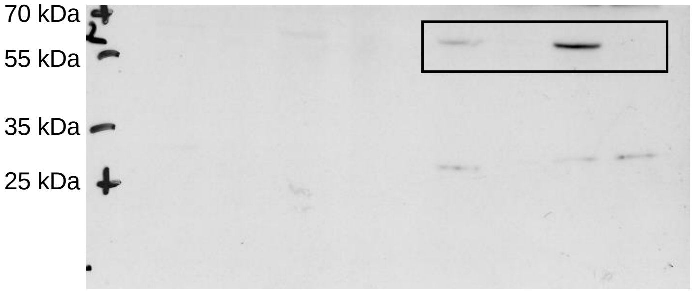

(2) Chk2

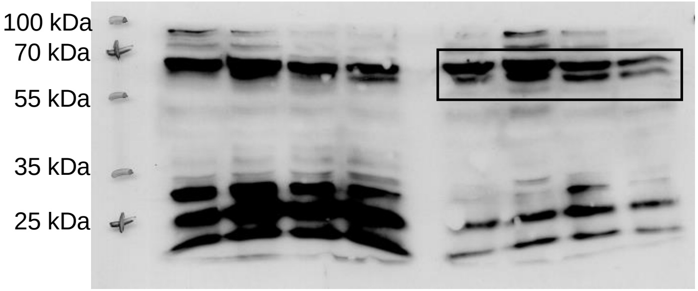

(3) GAPDH

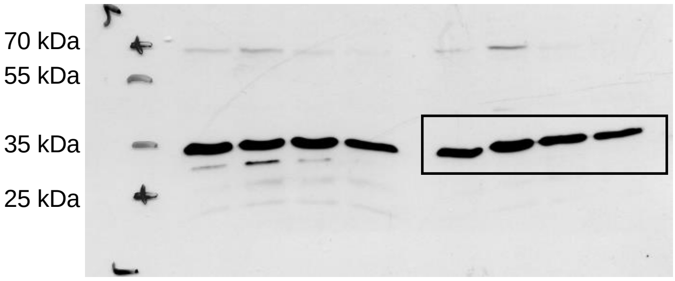

Figure 4 C – scanned original blots

HEK 293T – TRX1-CS -/+ Bleomycin/H_2O_2

(1) ATM, Vinculin

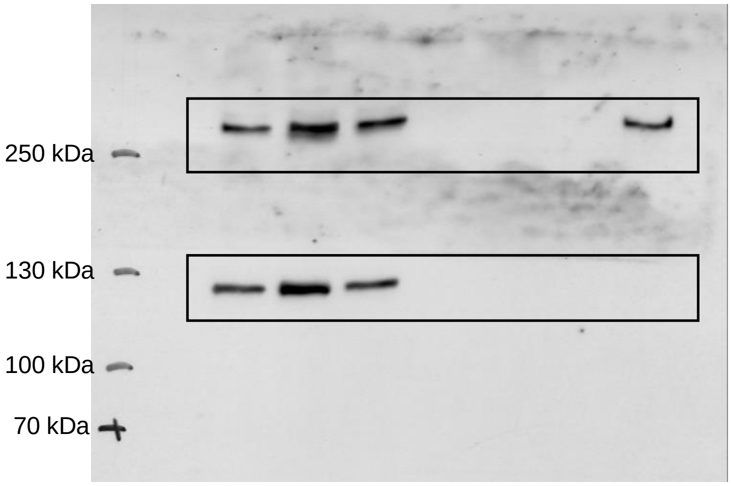

(2) PRDX1

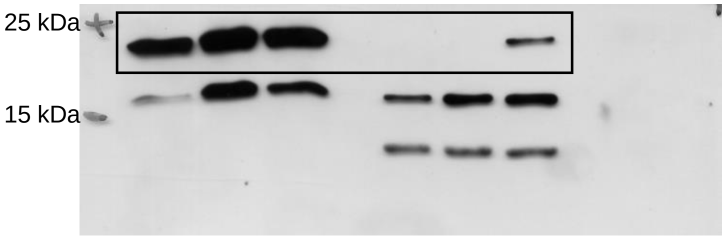

(3) His

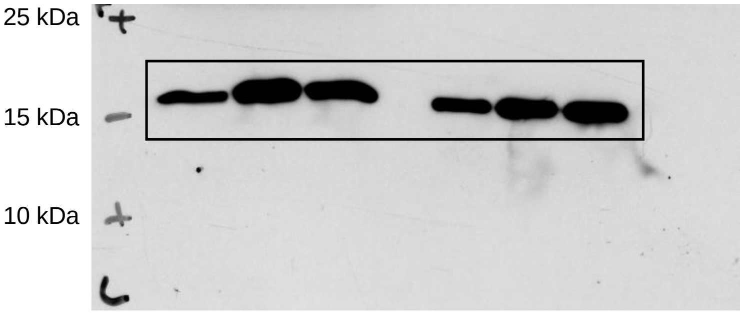

(4) β-Actin

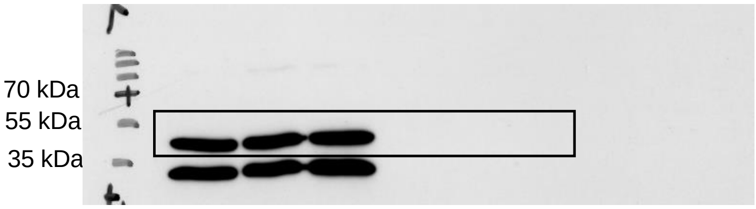

Figure S1 A – scanned original blots

HEK 293T – TRX1-CS -/+ H_2O_2

(1) ATM, Vinculin

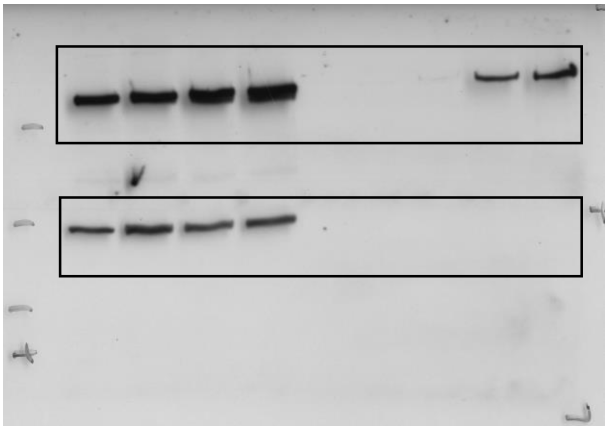

(2) PRDX1

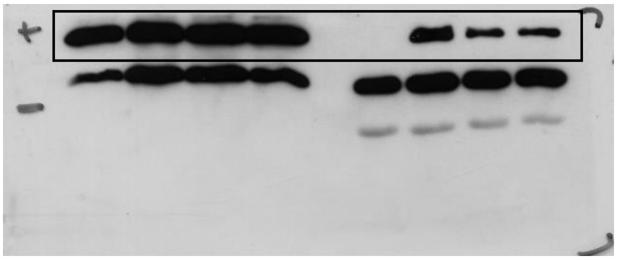

(3) His

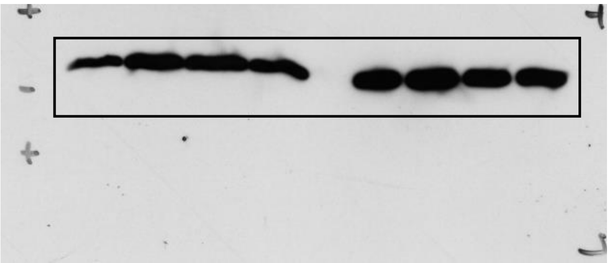

(4) β-Actin

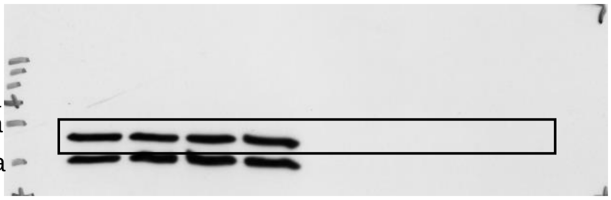

Figure S1 B – scanned original blots

HEK 293T – TRX1-CS -/+ KU55933/H_2O_2

(1) ATM, Vinculin

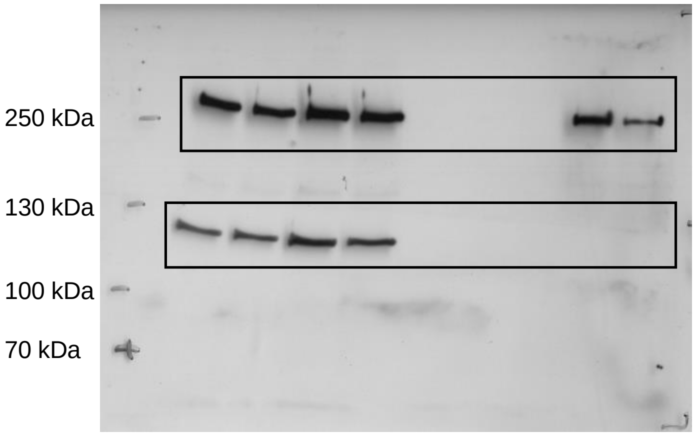

(2) PRDX1

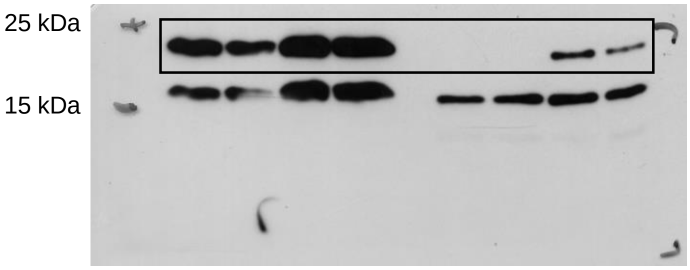

(3) His

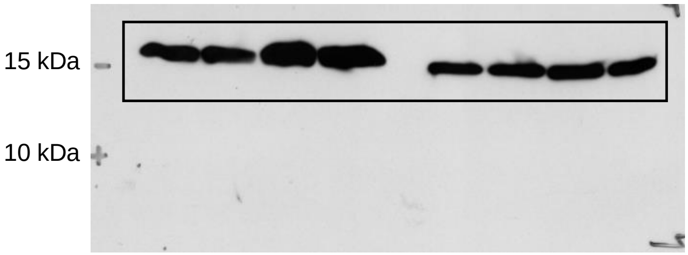

(4) β-Actin

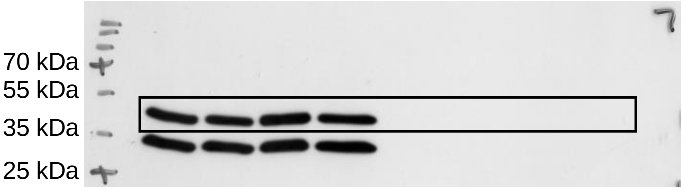

Supplement: S2 Fig — MLFs were treated for 24 h with Auranofin (3 μM) or left untreated. Cells were stained with PI and Hoechst and analyzed by fluorescent microscopy. Scale bar = 500 μm. (PDF) [file pone.0244060.s002.pdf]
